# Cryptosporidiosis Associated with Ozonated Apple Cider

**DOI:** 10.3201/eid1204.050796

**Published:** 2006-04

**Authors:** Brian G. Blackburn, Jacek M. Mazurek, Michele Hlavsa, Jean Park, Matt Tillapaw, MaryKay Parrish, Ellen Salehi, William Franks, Elizabeth Koch, Forrest Smith, Lihua Xiao, Michael Arrowood, Vince Hill, Alex da Silva, Stephanie Johnston, Jeffrey L. Jones

**Affiliations:** * Centers for Disease Control and Prevention, Atlanta, Georgia, USA;; † Ohio Department of Health, Columbus, Ohio, USA;; ‡ Atlanta Research and Education Foundation, Atlanta, Georgia, USA;; § Stark County Health Department, Canton, Ohio, USA

**Keywords:** Cryptosporidiosis, diarrhea, epidemiology, case-control, cohort, dispatch

## Abstract

We linked an outbreak of cryptosporidiosis to ozonated apple cider by using molecular and epidemiologic methods. Because ozonation was insufficient in preventing this outbreak, its use in rendering apple cider safe for drinking is questioned.

*Cryptosporidium* spp. are protozoan parasites transmitted by the fecal-oral route that cause prolonged diarrhea. Only 2 reports describe outbreaks associated with apple cider (*1**,**2*), and none have been associated with ozonated cider. In October 2003, a northeast Ohio health department identified 12 local residents with laboratory-confirmed cryptosporidiosis; 11 had drunk a locally produced, ozonated apple cider (cider A) in the 2 weeks before illness. The cider was embargoed on October 24, and we initiated an investigation by using epidemiologic and molecular techniques to determine the cause and extent of the outbreak and the role played by the cider and ozonation.

## The Study

We defined a probable case as a northeast Ohio resident with otherwise unexplained diarrhea for >3 days from September 1 to November 30, 2003, and a laboratory-confirmed case as a person with diarrhea and a positive *Cryptosporidium* laboratory result. Case finding encompassed interviewing persons with diarrhea who came to local health departments and emergency rooms and participants of school outings at which cider A was served. We then conducted 2 epidemiologic studies in which questionnaires showed exposures classically associated with *Cryptosporidium* transmission such as food, drinking and recreational water, person-to-person contact, animals, and travel.

Study 1 compared laboratory-confirmed case-patients and 2 controls (persons without diarrhea, abdominal pain, or vomiting) per case matched on age and county of residence and identified through random-digit dialing. Additionally, we conducted a retrospective cohort study of school children (study 2) who attended field trips at which cider A was served.

Stool samples from case-patients were screened by wet preparation and tested for *Cryptosporidium* by using an immunofluorescent assay (Meridian Merifluor *Cryptosporidium*/*Giardia* DFA kit, Meridian Bioscience, Cincinnati, OH, USA). Identification of *Cryptosporidium* in these samples was attempted by using 2 methods: 1) genotyping isolates by polymerase chain reaction–restriction fragment length polymorphism (PCR-RFLP) analysis of the small subunit (SSU) rRNA gene (*3**,**4*) with subtyping by DNA sequence analysis of the GP60 gene (*5*), and 2) amplification of the SSU rRNA gene with genotype differentiation by a microsatellite marker (ML-1) (*6**,**7*).

Cider samples were concentrated by centrifugation, and water samples were concentrated by Environmental Protection Agency method 1623 (*8*). *Cryptosporidium* genotyping was performed by using the same methods described for stool samples (*4*). Newly designed GP60 primers were used to subtype cider samples (*9*).

We identified 23 laboratory-confirmed and 121 probable case-patients with onset dates from September 3 to November 19, 2003 (Figure); the first cider-related case occurred September 22. The median patient age was 20 years (range 1–80). The median incubation period was 7 days (range 1–21), and median period of diarrhea was 7 days (range 3–52). Two patients were hospitalized and none died.

**Figure:**
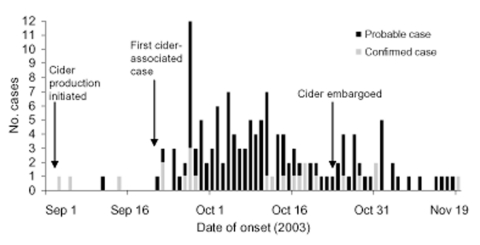
Laboratory-confirmed (n = 23) and probable (n = 121) cases of cryptosporidiosis from drinking ozonated apple cider, Ohio, 2003.

In study 1, we enrolled 19 laboratory-confirmed case-patients and 38 age-matched, community-based controls. Twelve of 19 case-patients, but 0 of 38 controls, had drunk cider A. Although the matched odds ratio (OR) for this association was incalculably high, the lower limit of the 95% confidence interval (CI) was 5.6. Although 3 other exposures were also associated with illness by univariate analysis (Table), only drinking cider A was associated with illness in a conditional logistic regression model that included all of these exposures (estimated OR 14.0, 95% CI 1.8–167).

**Table:** Association of selected exposures to cryptosporidiosis from drinking of ozonated apple cider, Ohio, 2003*

Exposure	Cases (n = 19), No. exposed/total (%)	Controls (n = 38), No. exposed/total (%)	MOR	95% CI
Drinking cider A	**12/19 (63)**	**0/38 (0)**	–	Lower limit **5.6**†
Eating apples from orchard A	**4/19 (21)**	**0/38 (0)**	–	Lower limit **1.3**†
Eating green onions	0/19 (0)	6/37 (16)	0	–
Eating lettuce or garden salads	9/18 (50)	26/38 (68)	0.6	0.2–1.6
Eating raw berries	0/19 (0)	9/38 (24)	0	–
Drinking cider other than cider A	9/19 (47)	9/37 (24)	3.3	0.9–11.6
Drinking unfiltered tap water at home	12/19 (63)	17/37 (46)	2.3	0.7–8.1
Drinking well water at home	6/18 (33)	11/38 (29)	1.1	0.4–3.6
Swimming in recreational water	1/19 (5)	7/38 (18)	0.3	0.03–2.2
Household contact in day care	1/19 (5)	4/37 (11)	0.3	0.02–5.3
Household contact with diarrhea	**12/19 (63)**	**7/38 (18)**	**5.3**	**1.5–18.4**
Travel >50 miles from home	7/19 (37)	12/38 (32)	1.3	0.4–4.9
Contact with any animal	14/19 (74)	33/38 (87)	0.4	0.08–1.7
Contact with cattle	**6/19 (32)**	**3/38 (8)**	**5.5**	**1.1–28.4**

*MOR, matched odds ratio; CI, confidence interval. Significant values are in **boldface**. †Exact one-sided 95% CI.

In study 2, we enrolled 402 persons who participated in outings at which cider A was served. Thirty-three (10%) of the 329 persons who drank cider A became ill, while only 2 (3%) of 73 who did not drink cider A became ill (adjusted relative risk 4.7, 95% CI 1.2–18.1). Only drinking cider A remained significantly associated with illness in a multivariate logistic regression model that included 4 other exposures that increased risk in univariate analysis (estimated OR 5.7, 95% CI 1.2–26.6).

No employees from the orchard or the separate cider pressing facility reported diarrhea from September 1 to November 7, 2003 (date of interview). The water supply for both was negative for *Cryptosporidium*; employees used "few" dropped apples for cider production. During production, an ozonating apparatus (Golden Buffalo Company, Orange, CA, USA) was used to treat the cider, which was then stored in refrigerated tanks. Most cider was ozonated a second time, then sold in plastic jugs on-site and at nearby grocery stores. Remaining cider was sold through a tap in the orchard's store. In contrast to jugged cider, this cider was not reozonated.

We performed a *Cryptosporidium* PCR on all 14 available samples from the laboratory-confirmed case-patients. Twelve (85.7%) of these were PCR positive; 11 of these 12 samples were identified as *Cryptosporidium parvum* and 1 as the cervine *Cryptosporidium* genotype (W4). Subtype identification of stool samples yielded 2 closely related *C*. *parvum* subtypes (IIaA15G2R1 and IIaA17G2R1).

The remaining contents of a jug of cider A that a laboratory-confirmed case-patient had partially drunk were also positive by PCR for *C*. *parvum* subtype IIaA17G2R1. This case-patient's stool sample yielded the same subtype of *C*. *parvum*, as did 4 other case-patients' stool samples; all of these persons drank cider A in the 2 weeks before illness onset.

## Conclusions

Our investigation strongly implicates cider A as the cause of this outbreak. The timing of cider A production closely paralleled the outbreak, and drinking cider A was the only predictor significantly associated with illness in both univariate and multivariate analyses of both epidemiologic studies. Furthermore, detection of *C*. *parvum* subtype IIaA17G2R1 from the sample of partially drunk cider A from a laboratory-confirmed case-patient provided further evidence of this link.

The 2 *C*. *parvum* subtypes found in this outbreak likely represent a common contamination source; both are common in cattle, and multiple subtypes are commonly found on farms (*10*). This outbreak highlights the need for continued development of molecular biologic methods because these techniques will be useful to identify and define future *Cryptosporidium* outbreaks and supplement epidemiologic associations.

An issue raised by this outbreak is the role of ozonation in the treatment of apple cider. New regulations (Hazard Analysis and Critical Control Point [HACCP] standards) enacted by the US Food and Drug Administration (FDA) in 2001 (*11*) require juice manufacturers to demonstrate a 5-log reduction of "the most resistant microorganism of public health significance" in their production process.

Because this is the third reported *Cryptosporidium* outbreak related to unpasteurized apple cider (*1**,**2*), whatever sterilization procedure is used must be effective against *Cryptosporidium*. Although pasteurization kills *Cryptosporidium* oocysts (*12*), no data exist on the use of ozonation against *Cryptosporidium* in food or juice products, where turbidity and low temperature render ozonation less effective (*13**,**14*). Furthermore, ozonation is difficult to standardize because effectiveness depends on contact time and concentration.

The consideration of ozonation is important because effective disinfection would have prevented the outbreak unless contamination occurred at the final step before distribution. Furthermore, of the 12 ill persons in the case-control study who drank cider A, 6 drank once-ozonated cider and 6 drank twice-ozonated cider, suggesting that even repeated ozonation was inadequate to kill *Cryptosporidium*. The failure of ozone could have been due to an inherent inadequacy for killing *Cryptosporidium* in apple cider or improper use; either possibility emphasizes the problems with ozonation in this setting and the need for further testing before its use is accepted.

Given the paucity of evidence supporting ozonation for apple cider disinfection or for killing *Cryptosporidium* in this product, and its apparent failure in this outbreak, the FDA issued an addendum to its HACCP rule (*15*). This addendum advises that juice makers should not use ozone in their manufacturing process unless they can prove a 5-log pathogen reduction through ozonation. To our knowledge, no studies have established this reduction to date.
